# The Zinc-Metallothionein Redox System Reduces Oxidative Stress in Retinal Pigment Epithelial Cells

**DOI:** 10.3390/nu10121874

**Published:** 2018-12-02

**Authors:** Sara Rodríguez-Menéndez, Montserrat García, Beatriz Fernández, Lydia Álvarez, Andrés Fernández-Vega-Cueto, Miguel Coca-Prados, Rosario Pereiro, Héctor González-Iglesias

**Affiliations:** 1 Instituto Universitario Fernández-Vega (Fundación de Investigación Oftalmológica, Universidad de Oviedo), 33012 Oviedo, Spain; rodriguezmenendez.sara@gmail.com (S.R.-M.); l.alvarez@fio.as (L.Á.); afvega89@gmail.com (A.F.-V.-C.); miguel.coca-prados@yale.edu (M.C.-P.); mrpereiro@uniovi.es (R.P.); h.gonzalez@fio.as (H.G.-I.); 2 Department of Physical and Analytical Chemistry, Faculty of Chemistry, University of Oviedo, Julián Clavería, 8, 33006 Oviedo, Spain; 3 Instituto Oftalmológico Fernández-Vega, Avda. Dres. Fernández-Vega, 34, 33012 Oviedo, Spain; 4 Department of Ophthalmology and Visual Sciences, Yale University School of Medicine, 300 George St, 8100A, New Haven, CT 06510, USA

**Keywords:** zinc, supplementation, metallothioneins, Zn-MT stoichiometry, retinal pigment epithelial cells, oxidative stress, mass-spectrometry

## Abstract

Oxidative stress affects all the structures of the human eye, particularly the retina and its retinal pigment epithelium (RPE). The RPE limits oxidative damage by several protective mechanisms, including the non-enzymatic antioxidant system zinc-metallothionein (Zn-MT). This work aimed to investigate the role of Zn-MT in the protection of RPE from the oxidative damage of reactive oxygen intermediates by analytical and biochemical-based techniques. The Zn-MT system was induced in an in vitro model of RPE cells and determined by elemental mass spectrometry with enriched isotopes and mathematical calculations. Induced-oxidative stress was quantified using fluorescent probes. We observed that 25, 50 or 100 μM of zinc induced Zn-MT synthesis (1.6-, 3.6- and 11.9-fold, respectively), while pre-treated cells with zinc (25, 50, and 100 μM) and subsequent 2,2′-Azobis(2-methylpropionamidine) dihydrochloride (AAPH) treatment increased Zn-MT levels in a lesser extent (0.8-, 2.1-, 6.1-fold, respectively), exerting a stoichiometric transition in the Zn-MT complex. Moreover, AAPH treatment decreased MT levels (0.4-fold), while the stoichiometry remained constant or slightly higher when compared to non-treated cells. Convincingly, induction of Zn-MT significantly attenuated oxidative stress produced by free radicals’ generators. We conclude that the stoichiometry of Zn-MT plays an important role in oxidative stress response, related with cellular metal homeostasis.

## 1. Introduction

The human eye is a highly specialized organ of vision in which the light energy interacts with the neurosensory cells of the retina, where the photoreception process occurs [1]. The retina consists of an inner multilayer of neurosensory cells and an outer single neuroepithelium, the retinal pigment epithelium (RPE). The RPE is a monolayer of pigmented cuboidal epithelial cells strongly polarized, playing a pivotal role in the visual cycle [2]. The main functions of the RPE include: the nourishment of photoreceptor cells, phagocytizing their outer segments; the transport of electrolytes, metabolites and vitamins between the neurosensory retina and the choroid, providing a selectively permeable barrier; the mediation of the immune response of the eye; and the absorption of light and reduction of light scatter within the eye, to decrease photo-oxidative stress [1,3,4,5]. 

The eye is constantly subjected to oxidative stress coming from multiple sources, including daily exposure to ultraviolet light, chemical insults and a highly oxidative milieu, inter alia [6]. The oxidative damage affects all the ocular structures, specifically those of the posterior pole, the neurosensory retina and the RPE. During ageing, RPE oxidative damage and inflammatory processes contribute to the pathogenesis of age-related eye diseases, including glaucoma, age-related macular degeneration (AMD), diabetic retinopathy and retinitis pigmentosa [7,8,9,10]. The continuous exposure of the RPE to light energy, the oxygen-rich environment and the high metabolic activity provide an ideal framework for the formation of reactive oxygen species (ROS) with the potential to damage proteins, deoxyribonucleic acid (DNA) and lipids [11]. 

Oxidative stress occurs from the formation of multiple ROS (superoxide, hydrogen peroxide, and hydroxyl radicals) promoting free radical production. The oxidative stress may be amplified by a continuing cycle of metabolic stress, tissue damage and cell death, leading to increased free radical production and compromise of the antioxidant capacity of free radical scavenger systems [12]. To limit the oxidative damage and maintain the structural integrity of the retina, the RPE have three lines of defense: (i) absorption and filtering of light through the specialized melanosomes pigments [13]; (ii) cellular repair systems of proteins, lipids and DNA [14]; and (iii) non-enzymatic (carotenoids, ascorbate, α-tocopherol, *β*-carotene, glutathione, metallothioneins, etc.) and enzymatic (superoxide dismutase, catalase, glutathione peroxidase, etc.) antioxidants neutralizing ROS [2].

An increase in oxidative stress along with a reduction in the protective mechanisms provided by antioxidant systems results in RPE dysfunction and death, compromising the neurosensory retina. The wide range of antioxidants contained in the RPE requires metal or semi-metals for their activity and/or function. An antioxidant system of increasing interest, both in the RPE and all the structures of the eye, is the zinc-metallothionein redox complex, which is involved in the protection against oxidative damage and inflammatory processes within the eye [15]. Metallothioneins (MTs) are small (6–7 kDa), metal-binding (Zn^2+^ and Cu^+^ are the biologically most relevant) and cysteine-rich (30%) cellular proteins [16]. In addition to defense against oxidative damage, they exhibit a diverse range of functions, such as neuroprotection, anti-inflammatory mediators and controlling cellular zinc homeostasis [17,18,19]. MTs fold in two separate domains: the α-domain with four Zn^2+^ and eleven Cys, and the *β*-domain with three Zn^2+^ and nine Cys (clusters with tetrahedral geometry) [20]. MTs are present in mammals in four isoforms (1 to 4), which are tissue specific and share a high degree of homology. MT1 (with 9 sub-isoforms) and MT2 are abundantly expressed in all type of tissues; MT3 is mainly present in brain and retina, whereas MT4 is found in stratified tissues [21]. 

In the human eye, MTs are highly expressed throughout the ocular tissues, particularly in the cornea, lens, retina and RPE, which are ocular natural barriers protecting against environmental insults [15]. The high distribution and diversity of MT isoforms in these tissues may be related to their continuous exposure to oxidative insults. Moreover, the role of MTs as zinc buffering contributes to the cellular defense [22]. In the RPE, MTs may act as protective mechanism against oxidative stress and induced apoptosis. The antioxidant function of MTs resides in the redox complex Zn-MT, which capture and neutralizes free radicals through cysteine sulfur ligands, serving as zinc-ion donors in a redox-dependent fashion [23,24,25]. 

In previous works, we studied the stoichiometry of the Zn-MT complex in three in vitro models of cells from eye tissues, corresponding with the main natural barriers of the eye that combat free radicals: the human corneal epithelial cells (HCEsv) [26], the human lens epithelial cells (HLEsv) [27], and the human retinal pigment epithelial cells (HRPEsv) [28]. We observed that the Zn-MT system exhibited different redox states that can be probably attributed to different antioxidant power. This distinct antioxidant capacity may be related to the roles of cornea, lens and RPE barriers protecting the eye against external insults. During AMD, the leading cause of irreversible blindness in western societies, there is a progressive degeneration of the RPE exacerbated by oxidative stress, affecting the health and integrity of photoreceptor cells [7,29]. Zn has been associated with AMD in major clinical studies [30,31,32]. The findings obtained in the AREDS study suggest that the use of antioxidants and zinc supplementation significantly reduce the progression of AMD. However, precisely how zinc supplementation helps to slow down the progression of this disease is not quite understood at the cellular and molecular levels, although zinc-supplementation has been associated with higher protection against RPE oxidation probably mediated by induction of MTs [33]. 

The question arises as to whether the induction of the Zn-MT system reduces oxidative stress in RPE cells, conferring higher protection against oxidative insults, and which is the mechanism underlying. We therefore aim to investigate the role of Zn-MT in protecting RPE cells from the oxidative damage of reactive oxygen intermediates. To this end, we used the in vitro cell culture model HRPEsv to examine the antioxidant response following induction of the Zn-MT. We applied analytical and biochemical-based techniques, including elemental mass spectrometry, to study the effects of zinc supplementation and oxidative stress induced by H_2_O_2_ and 2,2’-Azobis(2-amidinopropane) dihydrochloride (AAPH) on RPE cells. We observed that the Zn-MT system, induced by zinc sulphate, significantly reduced oxidative stress in RPE cultured cells, while the AAPH stressor decreased Zn-MT levels. The metal to MT ratio in the Zn-MT system may play a significant role in oxidative stress response, related with cellular metallostasis.

## 2. Materials and Methods 

### 2.1. Instrumentation

An inductively coupled plasma-mass spectrometer (ICP-MS) with double focusing magnetic sector field (Element 2 from Thermo Fisher Scientific, Bremen, Germany) was used, operating in medium resolution mode (R ≈ 4000) to remove polyatomic interferences of the sought isotopes. Setting experimental parameters, including Ar gas flow, torch position and ion lens voltage was daily optimized using a multi-element standard solution (1 ng·mL^−1^ Li, Co, Y, Ce and Tl, in 2% *w/w* HNO_3_) to obtain the best signal to noise ratio with minimal oxide-formation contribution (see Table S1 in electronic supplementary material). Determination of the Zn-MT system was carried out using an HPLC system (Thermo Finningan Surveyor from Thermo Fisher Scientific, Bremen, Germany) with the size exclusion chromatographic column Superdex^TM^ Peptide 10/300 GL (Healthcare Life Sciences, Little Chalfont, UK) and a Rheodyne six-port (Rohnert Park, CA, USA) fitted with a 50 µL sample loop, coupled online to the ICP-MS. A scavenger column (25 × 0.5 mm id) packed with Kelex-100 (Schering, Germany), placed between the pumps and the sample injector, was used to reduce the amount of metal ions in the mobile phase (see Table S1 in electronic supplementary material).

Fluorescence analyses to study oxidative stress in HRPEsv cells using commercial probes were performed on a Victor^TM^ X5 2030 multiplate reader spectrometer (Perkin Elmer, Turku, Finland). 

A 7500 Real Time (RT)-PCR System (Applied Biosystems Inc., Foster City, CA, USA) was used to quantify gene expression of MT isoforms.

### 2.2. Reagents and Materials

Zn, Cu and S standard solutions (1000 µg·mL^−1^) of natural isotopic abundances were purchased from Merck (Darmstadt, Germany). Certified isotopically enriched stable standards solutions of ^34^S (99.6% abundance), ^65^Cu (99% abundance) and ^67^Zn (89.6% abundance) were purchased from ISC-Science (Oviedo, Spain), while ^68^Zn (99.23% abundance) was obtained from Isoflex (San Francisco, CA, USA). The isotopically enriched zinc sulphate solution (^68^ZnSO_4_) was synthesized from the enriched tracer ^68^Zn, in the form of metal powder, by acidic digestion with suprapur H_2_SO_4_, at 110 °C for 150 min. Concentration and isotopic abundances of the synthesized ^68^ZnSO_4_ were determined by reverse isotopic dilution analysis (rIDA). All solutions were prepared with distilled deionized water (18.2 MΩ·cm^−1^) obtained from a Milli-Q water purification system (Millipore, Bedford, MA, USA). 

### 2.3. Experimental Procedures

#### 2.3.1. Human RPE Cell Line and Cultured Conditions 

A HRPEsv cell line, established in our laboratory from a primary culture of RPE cells obtained from the eyes of a 42-years-old donor (cadaver) and previously described [28], was used as in vitro model to study the protective effect of the Zn-MTs system against induced oxidative stress. 

Optimal culture conditions were established through cell viability assays. HRPEsv cells were seeded in 96-well plates, at a density of 3 × 10^3^ cell·well^−1^, in Dulbecco´s modified eagle medium/nutrient mixture F-12 (DMEMF12, 1% (*v/v*) penicillin/streptomycin and 10% (*v/v*) fetal bovine serum, 37 °C, 5% CO_2_). The next day, the cells were washed with PBS (pH 7.4) and cell culture medium was replaced by serum-free medium EX-CELL^®^ Hybridoma (Millipore Sigma, Burlington, MA, USA), supplemented with 10 mM glutamine and 1% (*v/v*) penicillin/streptomycin, for 24 h. Next, the cells were independently treated with the following reagents at concentrations ranged from: (i) 0 to 200 µM of ^68^ZnSO_4_, for 24 h; (ii) 0 to 100 mM of AAPH (98%, Acros Organics, Geel, Belgium), for 1, 2 and 24 h; and (iii) 0 to 5.5 mM H_2_O_2_ 30% (*w/w*) (Merck KGaA, Darmstadt, Germany), for 0.5 h; in triplicates. The viability of treated cells was determined post-assay using CyQUANT^®^ Cell Proliferation Assay Kit (Invitrogen^TM^, Carlsbad, CA, USA) as per manufacturer’s instructions, which produces fluorescence increase (λ_Ex/Em_ 485/535) upon binding to cellular nucleic acids, linearly correlated with cell viability.

#### 2.3.2. Gene Expression of MT Isoforms Following Zinc Supplementation

HRPEsv cells were either non-treated or treated in EX-CELL^®^ medium with: (i) 100 µM ^68^ZnSO_4_; (ii) 100 U·mL^−1^ interleukin-1α (IL1α; Millipore, Burlington, MA, USA), (iii) 120 U·mL^−1^ Erythropoietin (EPO; Merck, Darmstadt, Germany); (iv) 5 µM Lutein (Applichem, Darmstadt, Germany); or (v) 5µM Zexanthin (Cayman Chemical, An Arbor, MI, USA); for 24 h. Trypsinized cells were harvested and total RNA was isolated using mirVana^TM^ miRNA isolation kit (Invitrogen^TM^, Carlsbad, CA, USA). RNA quality and concentration was analyzed using Picodrop^TM^ spectrophotometer (Picodrop Limited, Hinxton, UK). Complementary DNA was obtained from 300 ng of total RNA through reverse transcriptase enzyme (High Capacity RNA-to-cDNA, Applied Biosystems, Foster City, CA, USA) and quantified by Picodrop^TM^. Gene expression of *MT2A*, *MT1F*, *MT1G* and *MT1X* isoforms was determined by quantitative real-time polymerase chain reaction (qRT-PCR) in a 7500 RT-PCR System (Applied Biosystems Inc., Foster City, CA, USA) and Taqman assays, using the following primers: *MT2A* Hs01591333_g1; *MT1F* Hs00744661_sH; *MT1G* Hs02578922_gH; and *MT1X* Hs00745167_sH; (Applied Biosystems Inc., Foster City, CA, USA). *GADPH* was used as endogenous control and each experiment was carried out in triplicate.

#### 2.3.3. Induction of Oxidative Stress in Cultured HRPEsv Cells

Two stressors were tested to induce oxidative stress in HRPEsv cells, in vitro: H_2_O_2_ (2 mM, 30 min-treatment) and AAPH (5 mM, 1 h-treatment). The levels of induced oxidative stress were determined using two fluorescent probes specific to reactive species: 2’-7’-dichlorofluorescein diacetate (DCF-DA) and Dihydrorhodamine 123 (DHR 123), both purchased from BioQuochem (Asturias, Spain). HRPEsv cells were seeded in 96-well plate (3 × 10^3^ cells·well^−1^) following the same conditions as before. Later, cells were pre-treated with 0, 25, 50, 100 µM of ^68^ZnSO_4_, or 10 mM of *N*-Acetyl-l-cysteine (NAC, Merck) in EX-CELL^®^ Hybridoma serum-free medium, for 24 h. Then, cultured cells were subsequent treated with either: (i) non-treated (control); (ii) treated with 2 mM of H_2_O_2_ for 30 min; or (iii) treated with 5 mM of APPH for 1 h. Finally, cells were washed with PBS and treated with 10 µM of each specific fluorescent probe, i.e., DCF-DA or DHR 123, according to the experiment. After incubation at 37 °C for 30 min, fluorescence intensity of DCF-DA probe (λ_Ex/Em_ 495/529 nm) or DHR 123 probe (λ_Ex/Em_ 500/536 nm) was measured in the Victor^TM^ X5 2030 microplate reader spectrometer following manufacturer’s instructions.

#### 2.3.4. Intracellular Localization of MTs by Conventional Immunocytochemistry

Cellular distribution of MT proteins in HRPEsv cells was visualized by indirect immunofluorescence. Immunocytochemistry of MTs was carried out under control conditions, after zinc supplementation (100 µM of ^68^ZnSO_4_, 24 h), oxidative stress induction (5 mM AAPH, 1 h), and the combination of both treatments (100 µM of ^68^ZnSO_4_ for 24 h + 5 mM AAPH for 1 h). Treated HRPEsv cells cultured on chamber slides (Thermo Fisher Scientific, Germany) were fixed with cold methanol (Merck Darmstadt, Germany), washed with PBS (3 times, 5 min) and permeated with 0.05% Tween 20 in phosphate buffered saline (PBS) for 10 min. Non-specific sites were blocked with goat serum (10% in PBS), and further incubated overnight with a mouse monoclonal antibody to MT1/2 (ab12228, Abcam; dilution 1:100), at 4 °C. Then, cells were washed with PBS (3 times, 5 min) and incubated at room temperature with Alexa 488 anti-mouse (Invitrogen; 1:500) for 1 h, and washed with PBS (3 times for 5 min). Nuclei were counterstained using 2 μg·mL^−1^ of 4’,6-diamidino-2-phenylindole (DAPI; Invitrogen). After washing in PBS and mounting in a solution of glycerol mounting medium (Dako, Agilent Technologies), the cellular distribution was examined by indirect immunofluorescence using a Leica TCS AOBS SP2 confocal microscope equipped with a 63 × /1.4 oil objective and 1.58 optical zoom (Leica Microsystems CMS GMBH, Wetzlar, Germany), at the “Unit of Photonic Microscopy and Image Processing” of the Scientific-Technical Services of the University of Oviedo.

#### 2.3.5. Quantification of Zn-MTs in HRPEsv Cells

Cellular treatments and soluble protein extraction. HRPEsv cells were seeded in T25 flasks (25 cm^2^ area) at a density of 2.5 × 10^5^ cell·flask^−1^ and cultured as previously described. Later, cultured cells were pre-treated with 0, 25, 50 and 100 µM of ^68^ZnSO_4_ in EX-CELL^®^ Hybridoma serum-free medium, for 24 h. Next, cells were either non-treated or treated with 5 mM of AAPH, for 1 h in triplicate. Treated HRPEsv cells were washed with PBS buffer, centrifuged at 200 g for 5 min to remove the supernatant and resuspended in 500 µL of 10 mM Tris-HCl (pH = 7.4) buffer to extract the cytosolic fraction containing the water-soluble proteins. Cellular membranes were broken down by ultra-sonication of the solution in three series of 10 KHz for 30 s on ice bath. After a centrifugation of 15,000× *g* at 4 °C for 10 min, supernatant containing Zn-MTs was collected, weighted, and stored at −80 °C (under N_2_ atmosphere to avoid cysteine-oxidation) for further quantification.

Quantification of the Zn-MT system by IPD-, *IDA- HPLC-ICP-MS.* The levels of zinc, MTs and the stoichiometry of the complex Zn-MT were determined through the quantification of Zn-, Cu- and S-binding proteins (including MTs) by HPLC-ICP-MS. The proteins and biomolecules contained in the biological samples under study were resolved using the SEC column, with 25 mM Tris-HCl base (pH 7.4) as mobile phase at 0.6 mL·min^−1^. After the chromatographic separation, the eluent of the column was on-line mixed with an isotopically enriched solution containing 0.15 ng·mL^−1^ of ^65^Cu, 15 ng·mL^−1^ of ^67^Zn and 300 ng·mL^−1^ of ^34^S, at 0.15 mL·min^−1^. The concentration of Cu- and S-binding proteins was determined by IDA, while the concentration of Zn-binding proteins was obtained applying the isotope pattern deconvolution (IPD) methodology, as previously described [26,27,28]. 

### 2.4. Statistical Analysis

Data are reported as mean ± standard deviation (SD) [34]. A *p*-value < 0.05 was considered statistically significant. Statistical analyses were performed using one-way analysis of variance (ANOVA) and Tukey’s post-hoc test using GraphPad InStat version 3.10 (GraphPad Software, La Jolla, CA, USA).

## 3. Results and Discussion

### 3.1. Optimizacion of HRPEsv Cell Culture Conditions

In order to define appropriate experimental conditions to induce MT synthesis and oxidative stress without cytotoxic effects, we first optimized cell culture conditions, including zinc, H_2_O_2_ and AAPH concentrations and incubation times. HRPEsv cells were used with passage number from 15 to 20. Cell viability for zinc treatment was determined at 24 h following the addition of ^68^ZnSO_4_ from 0 to 200 µM using CyQUANT cell proliferation kit (see Experimental section). Cell viability was also studied for AAPH and H_2_O_2_ stressors at concentrations ranged from 0 to 100 mM (at 0.5, 1 and 24 h) and from 0 to 5.5 mM (at 0.5 h), respectively. Fold-changes obtained from raw data and presented as percentage when comparing treated cells versus controls were plotted to obtain the survival rate for each condition assayed (data not shown). The maximum tolerable zinc sulphate concentration was established in 100 µM with survival rate above 80% We therefore settled the optimal zinc concentrations in 25, 50 and 100 µM for the Zn-MT induction experiments. The optimal H_2_O_2_ and AAPH concentrations were established in 2 mM (30 min) and 5 mM (1 h), respectively. The defined concentrations of zinc, H_2_O_2_ and AAPH were used in the subsequent experiments.

It should be noted that the use of EX-CELL^®^ Hybridoma culture medium was supplemented with glutamine and penicillin/streptomycin, but in the absence of fetal bovine serum during designed treatments to HRPEsv cells [28]. Fetal bovine serum contains serum albumin, aminoacids, growth factors, etc., owning a large zinc-binding capacity and affinity. Albumin and α-2-macroglobulin together bind more than 98% of zinc in culture the media. Moreover, aminoacids and other intrinsic components of the cell culture bind Zn to significant extent. As a result, only a small fraction of zinc in serum-supplemented medium is really available to be biologically active. Therefore, culture medium was not supplemented with fetal bovine serum while high concentrations of ^68^ZnSO_4_ were used to maintain optimum available zinc in our experiments and guarantee the synthesis of Zn-MTs [35,36].

### 3.2. MT Gene Expression in HRPEsv Following Zinc Supplementation

Aiming to confirm the effect of exogenously zinc on MT induction in HRPEsv cells, we carried out the MT genes expression analysis. Quantitative RT-PCR was used, in combination with Taqman probes, to determine the RNA expression levels of MT specific isoforms, i.e., *MT2A*, *MT1F*, *MT1G* and *MT1X*, before and after being exposed 24 h with 100 µM ^68^ZnSO_4_ (see Table S2 in electronic supplementary material). The cells were also treated with lutein, zeaxanthin, IL1α and EPO, separately for 24 h. The relative hybridization signal obtained for each of the MT isoforms was normalized with internal controls, and the obtained arbitrary units were converted into fold-change when comparing treatments against control. These isoforms were selected considering they are highly expressed in RPE [15]. As expected, MT expression was highly up-regulated by zinc (14 to 67 fold-change), indicating its great specificity to this element. When compared to other potential inductors, only the pro-inflammatory IL1α was capable to induce the MT expression in a lower extent. 

MTs can be induced in vivo by factors including zinc, glucocorticoids, cytokines and regulatory response elements in their genes [26,37,38]. We previously studied the mechanisms regulating MT gene expression and protein synthesis in the human eye [27]. While zinc and pro-inflammatory cytokines induce the up-regulation of MT expression, zinc itself inhibits cytokine expression, existing a crosstalk communication related to oxidative stress and inflammation. Zinc activates the metal regulatory transcription factor 1 (MTF-1), a zinc finger protein that binds to specific DNA motifs termed metal-responsive element, and therefore regulating the expression of MTs and other genes involved in metal homeostasis [39,40].

### 3.3. Induction of Zn-MT System Reduces Oxidative Stress in Cultured HRPEsv Cells

**Figure 1:**
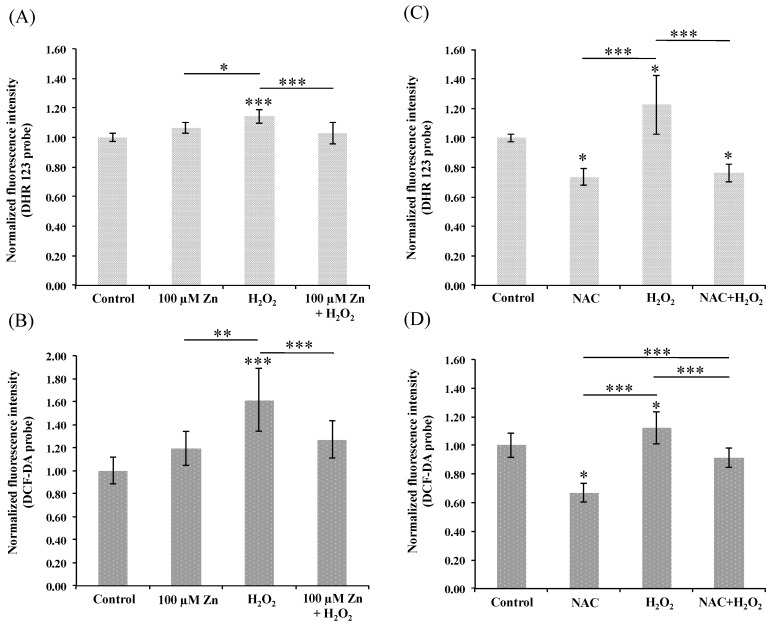
Histograms showing normalized fluorescence intensity of DHR 123 (panels **A** and **C**) and DCF-DA (panels **B** and **D**) probes in different treatment groups of HRPEsv cells, analyzed by a fluorescent plate reader. HRPEsv cells were either: (i) non-treated (see control bar in panels **A**–**D**); (ii) treated with H_2_O_2_ (2 mM for 30 min, see H_2_O_2_ bar in panels **A**–**D**); (iii) treated with zinc alone (100 μM for 24 h, see Zn bar in panels **A** and **B**); and (iv) pre-treated wither either 100 μM zinc for 24 h (see Zn + H_2_O_2_ bar in panels **A** and **B**) or (v) 10 mM NAC (see NAC + H_2_O_2_ bar in panels **C** and **D**) and followed in both cases by a treatment with H_2_O_2_ (2 mM for 30 min), respectively. Mean ± SD is plotted for 6 replicates for each condition. Data analyzed by one-way ANOVA followed by Tukey’s multiple comparison tests. * *p* < 0.05; ** *p* < 0.01; *** *p* < 0.001.

The in vitro HRPEsv cell culture model was used to study the effects of the oxidative stressors H_2_O_2_ or AAPH before or after the induction of Zn-MTs by zinc. The H_2_O_2_ stressor (2 mM, 30 min) was first used to induce oxidative stress in HRPEsv cells, and the fluorescent probes DCF-DA and DHR 123 were tested for the quantification of oxidative species formation. HRPEsv cells were pre-treated with 100 µM of zinc, in the form of ^68^ZnSO_4_ for 24 h, to induce the Zn-MT system and elicit whether is capable to reduce oxidative levels following H_2_O_2_ treatment. Moreover, NAC was used as positive control, since is a well-known reverser of induced oxidative stress [41]. NAC is an acetylated cysteine residue acting as free radical scavenging to lower endogenous oxidant levels and to protect cells against oxidative insults through different molecular mechanism including induction of glutathione (GSH) synthesis and its thiol-disulfide activity [42]. Figure 1 shows the histograms of the normalized fluorescence intensity (compared to control) using DHR 123 probe (panels A and C) or DCF-DA (panels B and D). ROS levels significantly increased (1.1- to 1.6-fold), after HRPEsv cells were treated with H_2_O_2_ (*p* < 0.001 panels A and B, and *p* < 0.05 panels C and D). Pre-treated cells with ^68^ZnSO_4_ for 24 h did not statistically significant increase ROS levels when comparing to control (panels A and B). Notably, pre-treated cells with 100 µM ^68^ZnSO_4_ for 24 h followed by H_2_O_2_ reduced significantly ROS levels when compared to H_2_O_2_ treated cells (*p* < 0.001, panels A and B). HRPEsv cells also were pre-treated with 25 or 50 µM ^68^ZnSO_4_ but obtained ROS levels did not exhibit statistically significant differences, probably because the H_2_O_2_ stressor provided high variability in inducing ROS, which may mask the variances of protection from zinc induction (data not shown). As expected, NAC treated-cells showed statistically significant lower levels of endogenous ROS when compared to control cells (*p* < 0.05, panels C and D) [42]. Although both probes worked properly, we decided to use in further experiments the DCF-DA considering the low detection limit obtained [43].

**Figure 2:**
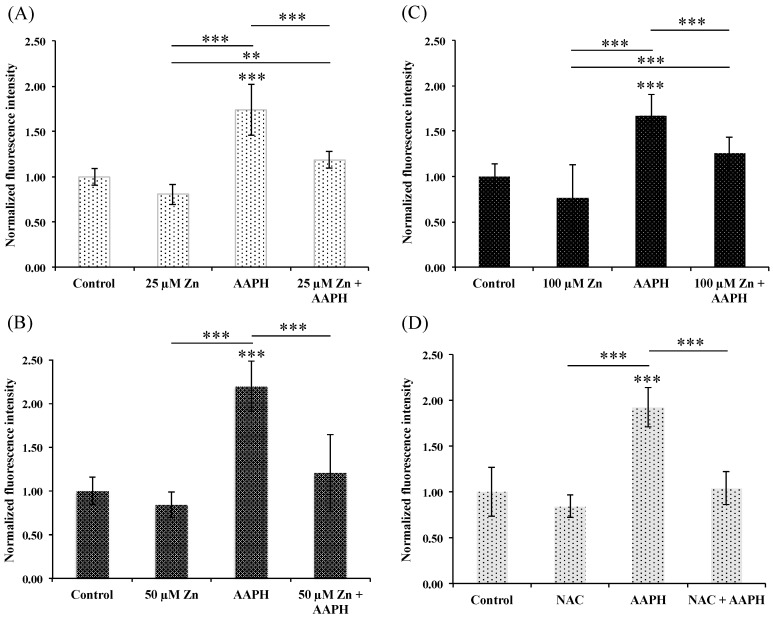
Histograms showing normalized fluorescence intensity of DCF-DA probe in different treatment groups of cells analyzed by a fluorescent plate reader. HRPEsv cells were either: (i) non-treated (see control bar in panels **A**–**D**); (ii) treated with AAPH (5 mM for 1 h, see AAPH bar in panels **A**–**D**); (iii) treated with 25, 50, 100 μM of zinc alone for 24 h (see Zn bar in panels, **A**–**C**) or with 10 mM of NAC alone for 24 h (see NAC bar in panels **D**); or (iv) pre-treated for 24 h with 25, 50, 100 μM of zinc or 10 mM of NAC, followed with a treatment with AAPH (5 mM for 1 h, see bar Zn+AAPH bar in panels **A**–**C** and see NAC+AAPH bar in panel **D**, respectively). Mean ± SD is plotted for 6 replicates for each condition. Data analyzed by one-way ANOVA followed by Tukey’s multiple comparison tests. * *p* < 0.05; ** *p* < 0.01; *** *p* < 0.001.

The high variability of free radical production by H_2_O_2_ is certainly related to the Fenton reaction, where iron catalyzes the conversion of hydrogen peroxide into hydroxyl radical intracellular, which turns out into a non-specific and unstable mechanism difficult to reproduce in vitro [43,44]. We therefore additionally optimized the use of the stressor AAPH to elicit free radicals in vitro and decipher our hypothesis. AAPH is a peroxyl radical generator, which decomposes spontaneously at physiological conditions producing constant rate of free radicals [45]. Figure 2 shows the histograms obtained using DCF-DA probe with fluorescence signal normalized with respect to non-treated cells. HRPEsv cells treated with 5 mM of AAPH for 1 h significantly increase the normalized fluorescence (i.e., oxidative stress) from 1.7- to 2.2-fold (*p* < 0.001, panels A, B, C and D) with highly reproducible values. To elucidate whether the induction of the Zn-MT system may reduce oxidative stress induced by AAPH, HRPEsv cells were pre-treated for 24 h with 25, 50 and 100 µM of ^68^ZnSO_4_ separately, using NAC (10 mM, 24 h) as positive control. Pre-treated cells with zinc did not increase ROS levels when compared to control (*p* > 0.5, panels A to C). Similarly, NAC pre-treated cells showed lower levels of endogenous ROS when compared to control cells, but no statistically significant (*p* > 0.5, panel D) probably as a consequence of the high variability of ROS levels observed in the non-treated cells. However, pre-treated cells with zinc (25, 50 and 100 µM) followed by AAPH stressor significantly reduced the ROS levels, when compared to AAPH treated cells (*p* < 0.001, panels A, B and C), with quantified free radical levels similar to controls (*p* > 0.5; 25, 50 or 100 µM Zn + AAPH vs. control). Additionally, HRPEsv cells treated with NAC diminished ROS levels when compared to AAPH treated cells with statistical significant differences (*p* < 0.001, panel D), while no differences were found when comparing NAC treated cells with cells pre-treated with NAC and subsequent AAPH treatment. 

In line with our results, Tate et al. [46] used an in vitro model of zinc deficiency to test the role of this element in protecting RPE cells from the oxidative damage of reactive oxygen intermediates induced by H_2_O_2_, indirectly by cell viability (MTT assay) and lipid peroxidation analysis. The RPE cells in reduced-zinc medium were less tolerant to oxidative stress when compared with normal zinc cells. They proposed that zinc-deficiency cells contained less MTs and other antioxidant enzymes, in which zinc may have an indirect protective role through the stabilization and protection of functional –SH groups from oxidative damage. Later, it was demonstrated that zinc treatment in the form of zinc-monocysteine increased antioxidant status in RPE cells and protected against H_2_O_2_ and *t*-butyl hydroperoxide stressor [47]. Moreover, Wood et al. [48] demonstrated that while low levels of zinc protect RPE cells from stress-induced effects, greater amounts might have the opposite role. Recently, Rajapakse et al. [49] studied the protective effect of zinc in oxidative stress-induced RPE death using oxidized photoreceptor outer segments and serum free conditions, suggesting that zinc supplementation protects cells by improving mitochondrial function and morphology and preventing lysosome rupture. 

It is well known that zinc is an essential element that serves as a catalytic cofactor to enzymes, folding proteins, and gene expression, but does not exhibit direct redox properties [50]. In vitro studies suggested that the antioxidant effect of zinc may at least be in part due to the specific induction of MTs [51]. Lu et al. [33] transfected cultured human D407 RPE cells with plasmids expressing MTs, observing an effective protection against oxidation induced by heme iron and apoptosis induced by UV light. Studies in vivo [52] suggested the key role of MTs in protection of neuronal retinal cells acting as endogenous antioxidants. MTs act as free radical scavengers, preventing lipid peroxidation and DNA damage. Besides, MTs are free radicals scavengers from different ROS including hydrogen peroxide, superoxide, nitric oxide and hydroxyl radicals [53]. Sulfhydryl groups of MTs react with hydroxyl radicals releasing zinc in a redox dependent cycle and taken up by the cellular membrane and the zincosomes [54]. The high content of sulfhydryl provides an affinity to free radicals more than 300-fold higher that of reduced glutathione [55]. We therefore aimed to further study the effects of zinc supplementation on the Zn-MT system under non-oxidative and oxidative stress conditions. 

### 3.4. Intracellular Distribution of MTs

We aimed to identify any preferential distribution of MTs on HRPEsv cells under oxidative stress conditions or zinc supplementation, by conventional immunocytochemistry. Figure S2 shows the cellular distribution of MTs in HRPEsv cultured cells obtained by indirect immunofluorescence using MT1/2 antibodies, under the following conditions: non-treated cells (panel A); 5 mM AAPH for 1 h (panel B); 100 μM zinc for 24 h (panel C); 100 μM zinc pre-treatment for 24 h followed by 5 mM AAPH for 1 h (panel D). Under control conditions, the MT staining was diffusely detected along the cytoplasm without any preferential distribution and in the nuclei with a dotted patter (panel A). HRPEsv cells treated with zinc (panel B), AAPH (panel C), or pre-treated with zinc and subsequent AAPH (panel D) stained both the cytoplasm and the cell nuclei with similar intensity when compared to non-treated cells. MTs are considered cytoplasmic proteins, mainly found in cellular cytoplasm and in some organelles, with lysosomal and mitochondrial localization [21]. However, nuclear localization of MTs has been also observed, probably through passive diffusion across the aqueous pore of the nuclear pore complex favored by their small molecular mass, or dependent on cytosolic factors and GTPases [56]. As published by Cherian et al. [57], MTs may protect the nucleus from oxidative damage and donate zinc to transcription factors, DNA synthesis proteins and metalloenzymes, controlling gene expression. We have observed nuclear localization of MTs both under control and stress conditions, without significant stain differences when comparing. Translocation of MTs from the cytoplasm to the nucleus occurs during the different phases of the cell cycle, specifically in cellular proliferation [53]. Therefore, the observed MT nuclei staining under control conditions may be explained by the actively proliferation of HRPEsv cells, hindering to identify any existing preferential nuclear translocation of MTs under oxidative stress conditions.

### 3.5. Induction of the Zn-MT System in HRPEsv Cells

We studied how zinc supplementation affects the Zn-MT redox system, and the further effects of oxidative stress-induced by AAPH on the levels of zinc, MTs, and the Zn-MTs stoichiometry, by IPD-, IDA-HPLC-ICP-MS following previous procedures [28].

#### 3.5.1. HRPEsv Cultured Cells Treated with Zinc

HRPEsv cells were either non-treated (control) or treated with 25, 50 and 100 µM of zinc in the form of ^68^ZnSO_4_, for 24 h. The levels of Zn-, Cu- and S- bound to MTs and to proteins other than MTs were quantified in the cytosolic fraction of HRPEsv cells by (SEC)-HPLC coupled to the sector field ICP-MS, using mathematical calculations based on IDA or IPD. Table 1 (see also Figure S3 in electronic supplementary material) shows the absolute concentration of zinc-binding proteins referred to total protein (μg·Zn·g^−1^ protein), i.e., Zn bound to MTs, to proteins other than MTs, and to all the zinc-binding proteins/molecules, in the non-treated and zinc treated cells. Concentration of total protein in the water-soluble protein fraction of HRPEsv was determined using BCA kits, with values ranging from 1.1 to 1.9 mg·mL^−1^. According to Table 1, the levels of zinc bound to MTs noticeably increased 3.8-, 13- and 51-fold under 25, 50 and 100 μM ^68^ZnSO_4_ treatments, respectively, when compared to control. However, the levels of other Zn-binding proteins slightly increased 1.4-, 2.0 and 3.0-fold following 25, 50 and 100 μM Zn supplementation, when compared to control. Zinc bound to MTs in non-treated cells accounts 43 ± 1% of total zinc bound to all species, while 25, 50 and 100 μM-treated cells reached 68 ± 16%, 83 ± 6% and 93 ± 1% of total zinc, respectively. Therefore, there is a specific induction of MTs at the protein level mediated by zinc, as previously reported [27,28]. The preferential binding site to transcription factors (MTF-1) stimulates transcription and translation into MT protein synthesis [39].

The levels of Cu- and S-binding proteins simultaneously quantified in the water-soluble protein fraction of HRPEsv cells were used to determine the MTs levels under control conditions and upon zinc treatments. To this end, the determined concentrations of Zn-, Cu- and S-bound to MTs provided the absolute concentration of MTs, as discussed elsewhere [28]. Table 2 (see also Figure S4) contains the concentration of MTs (in mg of MTs per g of protein), discriminating the natural contribution of Zn-MTs (^nat^MTs) from the exogenous contribution of ^68^Zn-MTs (^68^MTs) by IPD mathematical calculations [26], in the absence (control) or presence of ^68^ZnSO_4_. According to Table 2, HRPEsv culture cells treated with 25, 50, or 100 μM zinc presented a sustainable increase in total-MTs levels of 1.6- (1.01 ± 0.28 mg·MTs·g^−1^ total protein), 3.6- (2.21 ± 0.31 mg·MTs·g^−1^ total protein) and 11.9-fold (7.30 ± 0.18 mg·MTs·g^−1^ total protein), respectively, when compared to control (0.61 ± 0.02 mg·MTs·g^−1^ total protein). Furthermore, during stimulation with 25, 50, or 100 μM zinc, the pool of MTs reached 85 ± 27%, 91 ± 9% and 98 ± 2% of the exogenous one (^68^MTs), respectively. Interestingly we found a down-regulation of 0.27-, 0.33- and 0.26-fold in ^nat^MT proteins following 25, 50, or 100 μM of ^68^ZnSO_4_ supplementation, respectively. To determine the real percentage of newly synthesized MTs, the ionic exchange of ^68^Zn with ^nat^Zn within MTs proteins must be considered. In HRPEsv culture cells treated alone with zinc, the percentage of de novo MTs significantly increased according to treatment as follows: 41% increase of new MTs upon exposure to 25 µM of ^68^ZnSO_4_; 72% with 50 µM of ^68^ZnSO_4_; 91% with 100 µM of ^68^ZnSO_4_. 

However, the Zn-MT system is not fully characterized unless the metal:MTs stoichiometry is determined. The elemental stoichiometric composition of the MTs complexes was calculated by the determination of the S to metal (Zn and Cu) ratio, i.e., Zn:Cu:MT [26], under control conditions and upon treatments with ^68^ZnSO_4_, as shown in Table 2. In HRPEsv non-treated cells, the stoichiometry reached Zn_1.4_Cu_0.15_MT, similar to previous results [28]. Under exposure to 25-μM of zinc, we found an increase in the Zn ratio levels to Zn_3.1_Cu_0.16_MT, while upon 50 μM zinc treatment the stoichiometry changed to Zn_4.8_Cu_0.09_MT, and under 100 μM zinc treatment the MT proteins were completely load of zinc, Zn_7_Cu_0.02_MT. Therefore, the stoichiometry was highly dependent on the concentrations of zinc used, until saturation of zinc binding sites in MTs as expected. 

#### 3.5.2. HRPEsv Cultured Cells Treated with Zinc and Subsequent Oxidative-Stress Induction by AAPH

HRPEsv cells were either non-treated (control) or pre-treated with 0, 25, 50 and 100 µM of ^68^ZnSO_4_, independently for 24 h and then with 5 mM AAPH for 1 h. As stated above, we determined the levels of Zn-, Cu- and S- bound to MTs and to proteins other than MTs in the cytosolic fraction of HRPEsv cells. As shown in Table 3 and Figure S5, the levels of zinc bound to MTs in pre-treated cells with zinc (25, 50 and 100 μM ^68^ZnSO_4_) and treated with the AAPH stressor, were increased 3.5-, 12.5- and 20.5-fold, respectively, when compared to control. However, treated cells with the stressor AAPH alone for 1 h resulted in similar levels of zinc bound to MTs when compared to control (0.94-fold change). Conversely, the levels of other zinc-binding proteins decreased 0.5-, 1.0-, 0.9-fold and increased 2.9-fold following 0, 25, 50 and 100 μM Zn pre-treatment and AAPH stressor, respectively, when compared to control. Zinc bound to MTs in non-treated cells accounts 30 ± 7% of total zinc bound to all species, while 0, 25, 50 and 100 μM zinc pre-treated cells and AAPH stressor reached 45 ± 9%, 60 ± 7% and 99 ± 4% of total zinc, respectively.

The absolute concentration of MTs (in mg of MTs·g^−1^ protein) is shown in Table 4 (see also Figure S6). HRPEsv cells treated with AAPH experimented a down-regulation of 0.4-fold in MT synthesis (0.5 ± 0.1 mg MTs·g^−1^ total protein), when compared to control (1.1 ± 0.4 mg MTs·g^−1^ total protein). Similarly, HRPEsv cells pre-treated with 25 μM of zinc and then with AAPH presented a decrease in total-MTs of 0.8-fold (0.9 ± 0.4 mg MTs·g^−1^ total protein), but with similar values to those obtained in the previous experiment. The high variability of total-MTs in the non-treated cells warrants this observation. Conversely, HRPEsv culture cells pre-treated with 50, or 100-μM zinc for 24 h and then with AAPH presented a high increase in total-MTs of 2.1- (2.3 ± 0.2 mg MTs·g^−1^ total protein) and 6.1-fold (6.7 ± 0.5 mg MTs·g^−1^ total protein), respectively. The use of an enriched stable isotope of zinc permitted once again to differentiate the natural contribution of MTs from the newly synthesized (see Figure S6). Pre-treated cells with 25, 50, or 100 μM zinc and subsequent treatment with AAPH yielded 89 ± 44%, 91 ± 8% and 98 ± 5% of exogenous MTs (^68^MTs), respectively. These experimental values are quite similar to those obtained without the use of the oxidative stressor. As previous, we found a downregulation in ^nat^MT proteins under all the conditions assayed. Interestingly, in HRPEsv cells pre-treated with zinc and 24 h later treated with AAPH (5 mM, 1 h), the percentage of de novo MTs did not significantly increased upon exposure to 25 µM of ^68^ZnSO_4_, and significantly increased 55% under exposure with 50 µM of ^68^ZnSO_4_ and 84% with 100 µM of ^68^ZnSO_4_.

We fully studied the Zn-MT system obtaining their metal to MT ratio to determine whether oxidative stress may affect its stoichiometry and therefore the antioxidant power. As shown in Table 2, under control conditions, we obtained a stoichiometry of Zn_1.4_Cu_0.11_MT. HRPEsv cultured cells treated with AAPH for 1 h reached Zn_2.07_Cu_0.17_MT, and therefore a significant increase of bound zinc when compared to control. Under exposure to 25 μM of zinc and then to AAPH, we found an increase in the Zn ratio levels to Zn_4.2_Cu_0.13_MT, while upon 50 μM zinc pre-treatment and subsequent AAPH treatment the stoichiometry changed to Zn_5.9_Cu_0.6_MT, and under 100 μM zinc pre-treatment and AAPH treatment the MT binding sites were saturated by zinc, i.e., Zn_7.4_Cu_0.03_MT. Once again, the metal to MT ratio is dependent on the concentration of zinc used, while HRPEsv cells treated with APPH may influence the stoichiometry as well.

### 3.6. Does the Zn-MTs Redox System Reduce Oxidative Stress in HRPEsv Cultured Cells?

We demonstrated the specific induction of Zn-MTs following zinc supplementation, with a stoichiometric change depending on the concentration of zinc used. The MT family consists of multiple isoforms, which occur as a dynamic protein with different metal-to-protein ratios depending on the specific isoform and the state of the cells [58]. Zinc specifically induces MT synthesis activating the metal response element-binding MTF-1 [38,59], while may not alter significantly the levels of other components of the cell antioxidant defense system, such as glutathione, glutathione-S-transferase, glutathione peroxidase, catalase and superoxide dismutase [51]. However, zinc has been shown to induce catalase activity that may be regulated through the stimulation or SP-1 or other transcriptional response elements [60].

In addition, the zinc-mediated MTs up-regulation at protein and RNA levels resulted in decreased oxidative stress induced by AAPH and H_2_O_2_, in cultured HRPEsv cells. The intracellular pool of MTs is a mixture of oxidized (thionin), reduced (thionein) and zinc-bound (metallated) forms of the protein [61]. The dynamic equilibrium of these forms regulates zinc availability in response to intracellular ROS. MTs scavenge oxygen free radicals both in the metallated and the thionein form, through the oxidation of their multiple thionein groups. MTs contain twenty cysteine residues into two domains (*α* and *β*) with a low redox potential making it a preferential target for oxidation when compared with other free thiols [62]. The *β*-domain forms preferentially oxidative dimers being more easily unfolded than the *α*-domain with thiols accessible for redox reactions [63].

Although it has been speculated that MTs’ function as antioxidants is most effectively in the unstable thionein form (without the help of metal ions) [53], free radicals cause metal loss from protein and thiolate oxidation, suggesting that the thiolate groups of cysteine are the primary targets for the reaction of free radicals [64]. Actually, the MT affinity for zinc ions differs considerably, so that of the seven zinc-binding sites, three have a comparative weak metal binding affinity, which facilitates zinc release upon thiol oxidation. Therefore, the low stability constant of the three weakly bind zinc ions enables thermodynamically the release of zinc atoms from MT to other zinc-binding proteins, facilitating a more effectively interaction of thiols with free radicals [26]. Moreover, MTs transduce ROS signals into zinc signals that shape appropriate immune responses [19].

Despite this being an in vitro study, it is important to place the results in a physiological context. Our data suggest the importance of the Zn-MT stoichiometry to its antioxidant properties. We found a steady increase in the zinc to MT ratio upon 25, 50 and 100 μM zinc treatments, either with or without subsequent oxidative stress induction. However, the presence of the stressor AAPH, alone or following zinc pre-treatment, yielded an increase in the number of atoms of zinc bound to MTs, when compared with the absence of AAPH (Table 2 and Table 4). Interestingly, AAPH alone did not increase MT levels but induced a stoichiometric change (Zn_2.1_Cu_0.17_MT) with respect to control (Zn_1.4_Cu_0.11_MT). It must be stressed that when comparing HRPEsv cells pre-treated 24 h with 25, 50, and 100 μM ^68^ZnSO_4_, either with or without subsequent treatment with AAPH, the oxidative stressor reduced the Zn-MT protein levels. We infer that AAPH reduced the levels of MTs but increased the number of zinc atoms bound to MTs, which may be related with the antioxidant capacity of the Zn-MT system. Moreover, we hypothesized that saturated MTs (Zn_7_-MT) may have the greater antioxidant capacity, since when an excess of zinc is added the MTs may interact more effectively with ROS [27,59]. 

As previously reported, MTs are also inducible by acute response cytokines (IL-1 and TNFα) and glucocorticoids in RPE cells, indicating that MT could act as an acute response protein to protect retinal cells against oxidation incurred under stress [33]. Nevertheless, MT transcription may be regulated by oxidative stress according to seminal works [38,65,66], but distinct co-activators or signal transduction cascades are required to mediate MTF-1 activation of gene expression [40]. Moreover, according to Tate et al. [46] and to our results, induction of MT at mRNA level by oxidative stress does not entails a significant increase in MT protein synthesis, where oxidative stress decreased MT content and increase the number of zinc atoms per molecule of MT. 

### 3.7. Biological Implications of the Zn-MTs Antioxidant System in the RPE

The RPE defines the selectivity of the outer blood-retinal barrier, the cellular functions of which are critical for maintaining the nourishment and integrity of photoreceptor cells. The oxidative damage affects the RPE and the neurosensory retina, participating in the pathogenesis of eye diseases such as age-related macular degeneration and diabetic retinopathy [67]. MTs are part of the non-enzymatic lines of defense of the RPE against oxidative stress and apoptosis. The antioxidant properties of MT may be very important in the retina-RPE protecting against UV-induced oxidative stress by neutralizing free radicals [33] and muffling the influx of toxic substances [15].

The use of an in vitro model of cultured RPE cells provided a useful alternative to in vivo research, for which the access to fresh native human tissue is limited [68]. HRPEsv cells mimic some of the interactions that occur in vivo allowing the study of the Zn-MTs redox system and the effects of induced oxidative stress. However, two fundamental limitations of this model must be considered: the lack of full spectrum of signals from retina and choroid interacting with the RPE and the absence of melanin in melanosomes. Melanin is an effective antioxidant trapping stray light-induced free radicals from the photoreceptors to protect RPE cells [13]. The in vitro model used is a simplified system and therefore still need to be cautious when comparing the results obtained using cell culture to similar experiments using native tissue in vitro.

The protective effects of different antioxidants have been widely studied in RPE cells. While the phytochemical lutein may protect ARPE-19 cells from cytotoxic oxidative stress induced by H_2_O_2_, zeaxanthin did not show any cytoprotective effect [69]. In addition, curcumin (10 µM) decreased significantly oxidative stress in ARPE-19 cells, probably by interacting in the ARE/Nrf2 pathway [70]. However, the system Zn-MTs may have greater antioxidant capacity due to its high content of sulfhydryl groups [55] and the different metal binding affinity, facilitating zinc release upon oxidative stress conditions [26]. Under physiological oxidative conditions, zinc bound to MT is released through the thiolate cluster (forming the MT-disulfide), accumulated into zincosomes or in mitochondria, or be donated to other zinc-binding proteins. Under reduced conditions, zinc is quickly incorporated into the thionein, reconstituting the Zn-MT system [15]. The stoichiometry of Zn-MTs is therefore of fundamental importance to oxidative stress response and to transduce oxidative signals into zinc signals, regulating cellular metal homeostasis and transcription factors. Moreover, the redox properties of Zn-MTs are critical for buffering zinc ions, which modulates the cellular immune function of T lymphocytes via cytokine signaling [71,72,73]. Further understanding of the molecular mechanism related to Zn-MTs antioxidant properties, metabolism and zinc homeostasis would contribute the knowledge of the pathophysiology of eye diseases affecting the RPE.

## 4. Conclusions

We used an in vitro model of RPE cells to study the protective role of the Zn-MT system against oxidative stress. Zinc, in the form of zinc sulphate, highly upregulated the expression of MTs, both at mRNA and protein levels and significantly reduced oxidative stress in vitro. The oxidative stressor AAPH decreased Zn-MT levels, while the stoichiometry of the system slightly increased when compared to non-treated cells. Zn-MTs are known free radicals scavengers owing to their high content of sulfhydryl, providing high affinity to react with hydroxyl radicals releasing zinc in a redox dependent cycle. Therefore, the stoichiometry of the Zn-MT complex may play an important role in oxidative stress response, related with cellular metal homeostasis and transcription factors regulated by the redox chemistry of the complex.

Among the several limitations detected in this study, the HRPEsv in vitro model used must be stressed. The immortalized HRPEsv cells are a simplified system of the RPE, although they express specific markers of the native tissue. Therefore, primary cultures of differentiated cells should be approached, previous to in vivo studies, for a better understanding of the antioxidant response. On the other hand, zinc supplementation was carried out under short-time treatment conditions (i.e., 24 h), just to induce MT synthesis, and therefore long-term conditions should be tested to study how zinc supplementation may exert beneficial effects under oxidative stress conditions. Another limitation of this work deals with the induction of the system Zn-MT. Although a consistent upregulation of Zn-MT was observed, the transfection of cultured cells with plasmids expressing MTs, without using zinc as inductor, may help to confirm the role of Zn-MTs protecting against oxidative stress. Moreover, future studies must address the link between cellular stress and the redistribution of zinc within the cell, determining whether oxidative stress triggers a surge in free Zn^2+^ that evokes zinc signaling.

## Figures and Tables

**Table 1:** Concentration of ^nat^Zn, ^68^Zn and total Zn (^nat^Zn + ^68^Zn) (in μg·Zn·g^−1^ protein) found in Zn binding-proteins/molecules, i.e., MTs, other than MTs and all Zn-binding proteins/molecules, in the water-soluble protein fraction of HRPEsv cells by ID-, IPD-SEC-ICP-MS. HRPEsv cells were either not treated (control) or exposed to ^68^ZnSO_4_ at 25, 50 or 100 μM, separately for 24 h.

Treatment	Zn-Binding Proteins	^nat^Zn	^68^Zn	Total Zn (^nat^Zn + ^68^Zn)
		(μg·g^−1^ Protein)	(μg·g^−1^ Protein)	(μg·g^−1^ Protein)
**Control**	Zn-MTs	8.9 ± 0.2	0.00 ± 0.00	8.9 ± 0.2
	Other Zn-binding proteins	11.4 ± 0.4	0.00 ± 0.00	11.4 ± 0.4
	All Zn binding proteins/molecules	20.3 ± 0.6	0.00 ± 0.00	20.3 ± 0.6
**25 µM ^68^Zn**	Zn-MTs	5.7 ± 0.3	28.2 ± 8.4	33.9 ± 8.7
	Other Zn-binding proteins	6.6 ± 0.1	9.03 ± 0.03	15.6 ± 0.1
	All Zn binding proteins/molecules	12.3 ± 0.4	37.2 ± 8.4	49.6 ± 8.8
**50 µM ^68^Zn**	Zn-MTs	10.4 ± 5.0	104.0 ± 13.5	114.4 ± 18.5
	Other Zn-binding proteins	8.3 ± 0.9	14.8 ± 3.5	23.1 ±4.4
	All Zn binding proteins/molecules	18.6 ± 5.9	118.8 ± 17.0	137.5 ± 22.9
**100 µM ^68^Zn**	Zn-MTs	10.2 ± 0.4	441.8 ± 2.1	452.0 ± 2.5
	Other Zn-binding proteins	5.3 ± 0.3	29.7 ± 1.4	35.0 ± 1.6
	All Zn binding proteins/molecules	15.5 ± 0.7	471.5 ± 3.5	487.0 ± 4.1

**Table 2:** Concentration of MTs (in mg of MTs per g of protein) labeled with ^nat^MTs (natural contribution of zinc), ^68^MTs (exogenous contribution of zinc) and Total-Zn-MTs (sum of both natural and exogenous contributions), in HRPEsv cells. Cells were cultured for 24 h in the absence (control) or presence of ^68^ZnSO_4_ (at 25, 50 or 100 μM). The concentration of MTs containing ^nat^Zn and ^68^Zn was determined by ID-,IPD-SEC-ICP-MS. Total protein level (in mg) in HRPEsv cell extracts was determined by BCA assay kit. The elemental stoichiometric composition of the MTs complexes was calculated by the determination of the S to metal (Zn and Cu) ratio, i.e., Zn:Cu:MT.

Treatment	^nat^MTs (mg·g^−1^)	^68^MTs	^Total^MTs (mg·g^−1^)	Zn:Cu:MT Stoichiometry
	(Fold-Change) ^a^	(mg·g^−1^)	(Fold-Change) ^a^	
**Control**	0.61 ± 0.02 (1)	0.00 ± 0.00	0.61 ± 0.02 (1)	1.4 ± 0.1:0.15 ± 0.02:1
**25 µM ^68^Zn**	0.169 ± 0.001 (0.27)	0.84 ± 0.28	1.01 ± 0.28 (1.66)	3.1 ± 0.1:0.16 ± 0.03:1
**50 µM ^68^Zn**	0.20 ± 0.10 (0.33)	2.01 ± 0.21	2.21 ± 0.31 (3.62)	4.8 ± 0.1:0.09 ± 0.02:1
**100 µM ^68^Zn**	0.16 ± 0.01 (0.26)	7.13 ± 0.17	7.30 ± 0.18 (11.97)	7 ± 1:0.07 ± 0.02:1

^a^ fold-change showed in brackets, with respect to control.

**Table 3:** Concentration of ^nat^Zn, ^68^Zn and total Zn (^nat^Zn + ^68^Zn) (in μg Zn·g^−1^ protein) found in Zn binding-proteins/molecules, i.e., MTs, other than MTs and all Zn-binding proteins/molecules, in the water-soluble protein fraction of HRPEsv cells by ID-, IPD-SEC-ICP-MS. HRPEsv cells were either not treated (control) or exposed to ^68^ZnSO_4_ at 25, 50 or 100 μM, separately, for 24 h following treatment with 5 mM of AAPH for 1 h.

Treatment	Zn-Binding Proteins	^nat^Zn	^68^Zn	Total Zn (^nat^Zn + ^68^Zn)
		(μg·g^−1^ Protein)	(μg·g^−1^ Protein)	(μg·g^−1^ Protein)
**Control**	Zn-MTs	12.0 ± 2.8	0.00 ± 0.00	12.0 ± 2.8
	Other Zn-binding proteins	28.3 ± 9.5	0.00 ± 0.00	28.3 ± 9.5
	All Zn binding proteins/molecules	40.3 ± 12.3	0.00 ± 0.00	40.3 ± 12.3
**AAPH**	Zn-MTs	11.2 ± 2.4	0.00 ± 0.00	11.2 ± 2.4
	Other Zn-binding proteins	13.5 ± 0.1	0.00 ± 0.00	13.5 ± 0.1
	All Zn binding proteins/molecules	24.7 ± 2.5	0.00 ± 0.00	24.7 ± 2.5
**25 µM ^68^Zn + AAPH**	Zn-MTs	6.9 ± 0.8	34.5 ± 5.6	41.4 ± 6.4
	Other Zn-binding proteins	9.0 ± 3.2	18.2 ± 11.6	27.2 ± 14.8
	All Zn binding proteins/molecules	15.9 ± 4.0	52.6 ± 17.2	68.5 ± 21.2
**50 µM ^68^Zn + AAPH**	Zn-MTs	17.5 ± 0.5	131.9 ± 4.0	149.4 ± 4.5
	Other Zn-binding proteins	7.9 ± 0.3	17.6 ± 0.8	25.5 ± 1.2
	All Zn binding proteins/molecules	25.4 ± 0.9	149.5 ± 4.8	174.9 ± 5.7
**100 µM ^68^Zn + AAPH**	Zn-MTs	16.2 ± 0.4	532.3 ± 16.3	548.5 ± 15.9
	Other Zn-binding proteins	6.9 ± 0.9	50.4 ± 33.2	57.4 ± 34.2
	All Zn binding proteins/molecules	23.1 ± 1.3	582.7 ± 49.5	605.9 ± 50.8

**Table 4:** Concentration of MTs (in mg of MTs per g of protein) labeled with ^nat^MTs (natural contribution of zinc), ^68^MTs (exogenous contribution of zinc) and Total-Zn-MTs (sum of both natural and exogenous contributions), in HRPEsv cells. Cells were cultured for 24 h in the absence (control) or presence of ^68^ZnSO_4_ (at 25, 50 or 100 μM) following exposure to 5 mM of AAPH for 1 h. The concentration of MTs containing ^nat^Zn and ^68^Zn was determined by ID-,IPD-SEC-ICP-MS. Total protein level (in mg) in HRPEsv cell extracts was determined by BCA assay kit. The elemental stoichiometric composition of the MTs complexes was calculated by the determination of the S to metal (Zn and Cu) ratio, i.e., Zn:Cu:MT.

Treatment	^nat^MTs (mg·g^−1^)	^68^MTs	^Total^MTs (mg·g^−1^)	Zn:Cu:MT Stoichiometry
	(Fold-Change) ^a^	(mg·g^−1^)	(Fold-Change) ^a^	
**Control**	1.1 ± 0.4 (1)	0.00 ± 0.00	1.1 ± 0.4 (1)	1.4 ± 0.1:0.11 ± 0.042:1
**AAPH**	0.5 ± 0.1 (0.45)	0.00 ± 0.00	0.5 ± 0.1 (0.45)	2.07 ± 0.05:0.17 ± 0.04:1
**25 µM ^68^Zn + AAPH**	0.16 ± 0.02 (0.14)	0.8 ± 0.4	0.9 ± 0.4 (0.82)	4.2 ± 1.1:0.13 ± 0.03:1
**50 µM ^68^Zn + AAPH**	0.27 ± 0.02 (0.24)	2.1 ± 0.2	2.3 ± 0.2 (2.09)	5.9 ± 0.6:0.15 ± 0.01:1
**100 µM ^68^Zn + AAPH**	0.15 ± 0.08 (0.14)	6.6 ± 0.4	6.7 ± 0.5 (6.09)	7.4 ± 0.2:0.03 ± 0.01:1

^a^ fold-change showed in brackets.

## Supplementary Materials

The following are available online at http://www.mdpi.com/2072-6643/10/12/1874/s1, Figure S1: cell viability, Figure S2: cellular distribution of MT1/2 in HRPEsv cells by confocal microscopy, Figure S3: zinc levels in non-treated and zinc-treated cells, Figure S4: Levels of MTs in non-treated and zinc-treated cells, Figure S5: zinc levels in non-treated or zinc pre-treated and subsequent APPH treated cells, Figure S6: Levels of MTs in non-treated or zinc pre-treated and subsequent APPH treated cells, Table S1: ICP-MS instrumental operating conditions, Table S2: MT specific isoforms gene expression.
