# Supplementary material for: The Zinc-Metallothionein Redox System Reduces Oxidative Stress in Retinal Pigment Epithelial Cells
Source: Nutrients. 2018 Dec 2;10(12):1874. doi: 10.3390/nu10121874 (PMC6315569; doi:10.3390/nu10121874)
Supplement: Supplementary file 1 [file nutrients-10-01874-s001.pdf]

nutrients

Article—Supplementary Material

The zinc-metallothionein redox system reduces oxidative stress in retinal pigment epithelial cells

Sara Rodríguez-Menéndez[1,2], Montserrat García[1,3,*], Beatriz Fernández[1,2,*], Lydia Álvarez[1], Andrés Fernández-Vega-Cueto[1], Miguel Coca-Prados[1,4], Rosario Pereiro[1,2] and Héctor González-Iglesias[1,2,3]

1 Instituto Universitario Fernández-Vega (Fundación de Investigación Oftalmológica Fernández-Vega, Universidad de Oviedo), Spain; l.alvarez@fio.as (L.A.) Affiliation 1; e-mail@e-mail.com

2 Department of Physical and Analytical Chemistry, Faculty of Chemistry, University of Oviedo, Julián Clavería, 8, 33006, Oviedo, Spain; rodriguezmenendez.sara@gmail.com (S.R.M.); fernandezbeatriz@uniovi.es (B.F); mrpereiro@uniovi.es (R.P.); gonzalezhector@uniovi.es (H.G.I.)

3 Instituto Oftalmológico Fernández-Vega, Avda. Dres. Fernández-Vega, 34, 33012, Oviedo, Spain; mgarcia@fio.as (M.G.); h.gonzalez@fio.as (H.G.I.)

4 Department of Ophthalmology and Visual Sciences, Yale University School of Medicine, 300 George St, 8100A, New Haven, CT. 06510, USA; miguel.coca-prados@yale.edu (M.C.P.)

* Correspondence: mgarciadiaz@fio.as, Tel.: +34-985-24-0141; fernandezbeatriz@uniovi.es , Tel.: +34-985-10-3524

Received: date; Accepted: date; Published: date

Abstract: The Supplementary Material includes the supplemental information of optimal instrumental settings for ICP-MS analysis, the MT specific isoforms gene expression in HREPsv cells, the concentration of zinc in MTs, in proteins/biomolecules other than MTs, and in all zinc-binding proteins/biomolecules, and the concentration of MTs (^{nat}Zn-MTs and ^{68}Zn-MTs), obtained by IDA-, IPD-, SEC (HPLC)-ICP-MS, under selected experimental conditions.

1. Tables

Table S1. ICP-MS instrumental operating conditions. Data acquisition parameters and optimized chromatographic conditions for Zn-MT quantification by HPLC-ICP-MS analysis.

**Sector Field ICP-MS (Element 2)**		
Plasma parameters	RF power (W)	1295
	Cooling gas flow rate (L·min^{-1})	15.94
	Sample gas flow rate (L·min^{-1})	0.80
	Auxiliary gas flow rate (L·min^{-1})	0.88
Data acquisition parameters	Acquisition mode	Time resolved analysis
	Monitored isotopes	^{32,33,34}S, ^{64,66,67,68,70}Zn, ^{63,65}Cu
	Resolution	Medium (R~4000)
**Chromatographic Conditions**		
SEC-HPLC	Size exclusion column	Superdex™ Peptide 10/300 GL (MW range: 100-7000 Da)
	Mobile phase	25 mM Tris/HCl pH=7.4
	Flow rate (mL·min^{-1})	0.6
	Injection volume (µL)	50

Table S2. MT specific isoforms gene expression. MT specific isoforms gene expression in HRPEsv before (control) and after being exposed independently to the following reagents, for 24 h: i) 100 µM

$^{68}ZnSO_4$; ii) 100 U·mL^{-1} interleukin-1α (IL1α); iii) 120 U·mL^{-1} Erythropoietin (EPO); iv) 5 μM Lutein; v) 5 μM Zeaxanthin. The relative hybridization signal obtained for each of the MT isoforms was normalized with internal controls, and the obtained arbitrary units converted into fold-change when comparing treatments against control.

Gene	MT2A	MT1F	MT1G	MT1X
**Control**	1	1	1	1
**100 μM $^{68}ZnSO_4$**	14	20	67	28
**100 U·mL^{-1} IL1α**	4	2	3	2
**120 U·mL^{-1} EPO**	1	1	1	1
**5 μM Lutein**	1	2	1	1
**5 μM Zeaxanthin**	0.9	1	1	0.8

2. Figures

Figure S1. Cell viability. Percentage of relative cell viability depending upon zinc concentration (0 to 200 μM of Zn for 24 h). Mean ± SD is plotted for 5 replicates for each condition.

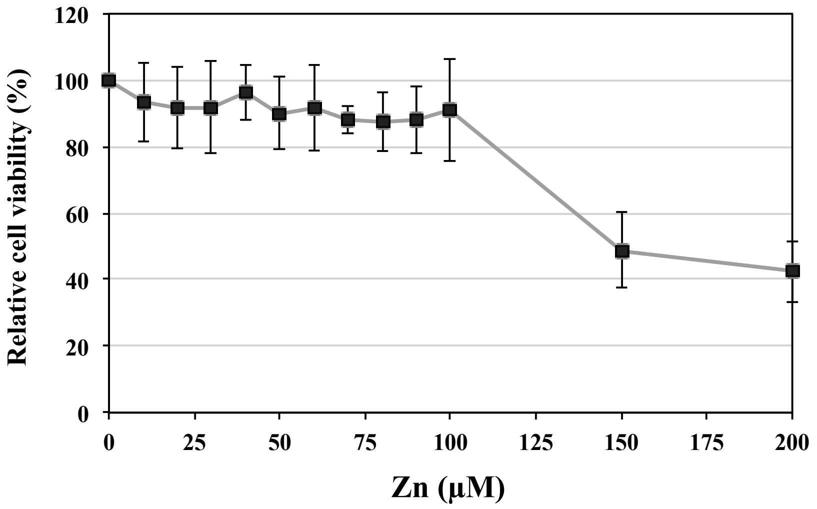

Figure S2. Cellular distribution of MT1/2 in HRPEsv cells by confocal microscopy. Columns: DAPI staining of cell nuclei micrographs (left); Alexa 488-labeled micrograph (middle); and merged image (right). Rows: A) Control; B) 100 μM zinc treatment ($^{68}ZnSO_4$, for 24 h); C) 5 mM AAPH treatment (1 h); D) 100 μM zinc ($^{68}ZnSO_4$) pre-treatment for 24 h followed by AAPH treatment (5mM for 1 h). Scale bar 25 μm.

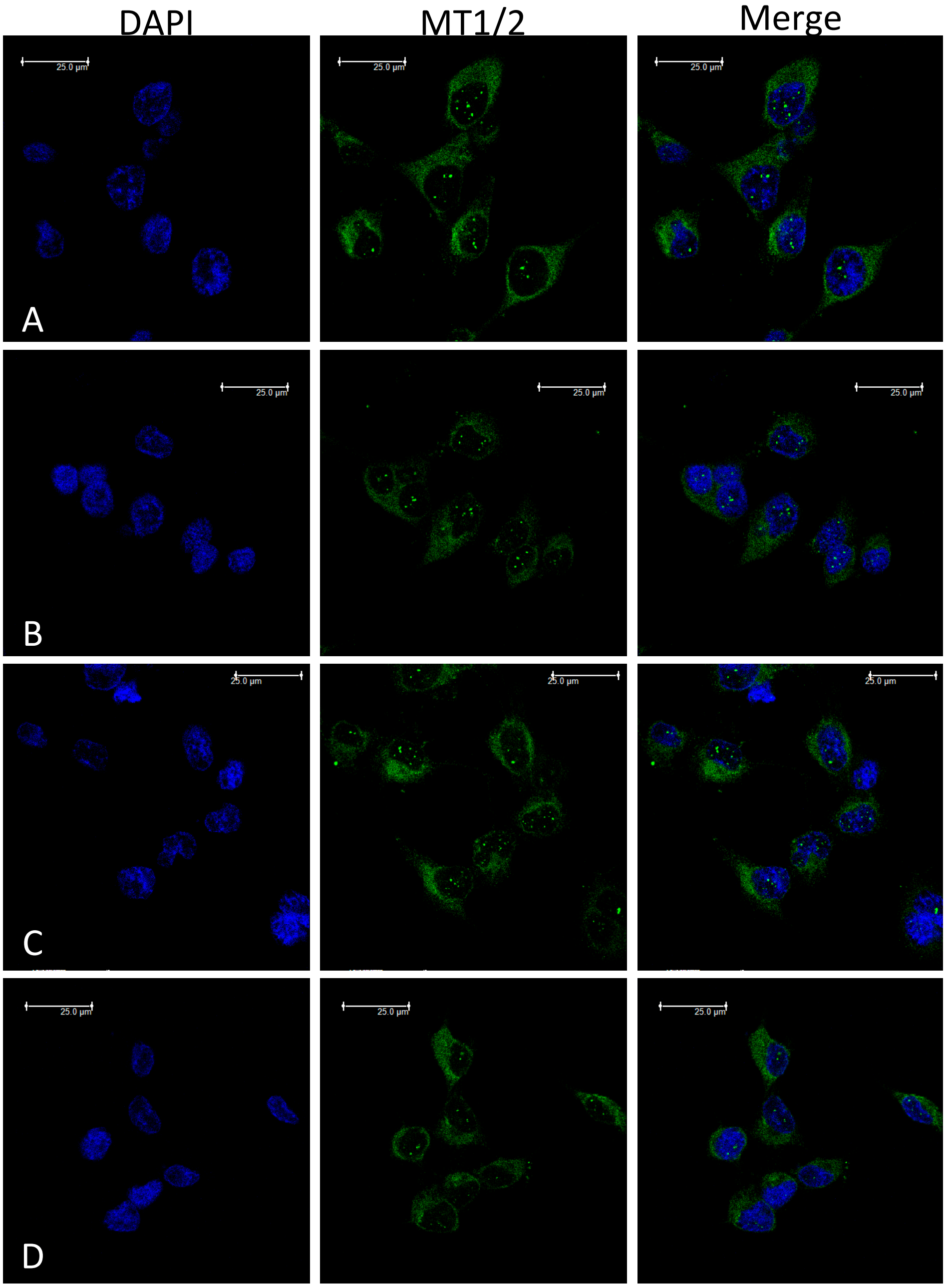

Figure S3. Zinc levels in non-treated and zinc-treated cells. Concentration of zinc (μg Zn·g^{-1} protein) in MTs (black bars dotted), in proteins/biomolecules other than MTs (white bars dotted), and in all Zn-binding proteins/biomolecules (grey bars dotted) obtained by IDA-, IPD-, SEC (HPLC)-ICP-MS in the water-soluble proteins in HRPEsv cells not treated (control) or following exposure to $^{68}ZnSO_4$ at 25, 50, or 100 μM, for 24 h.

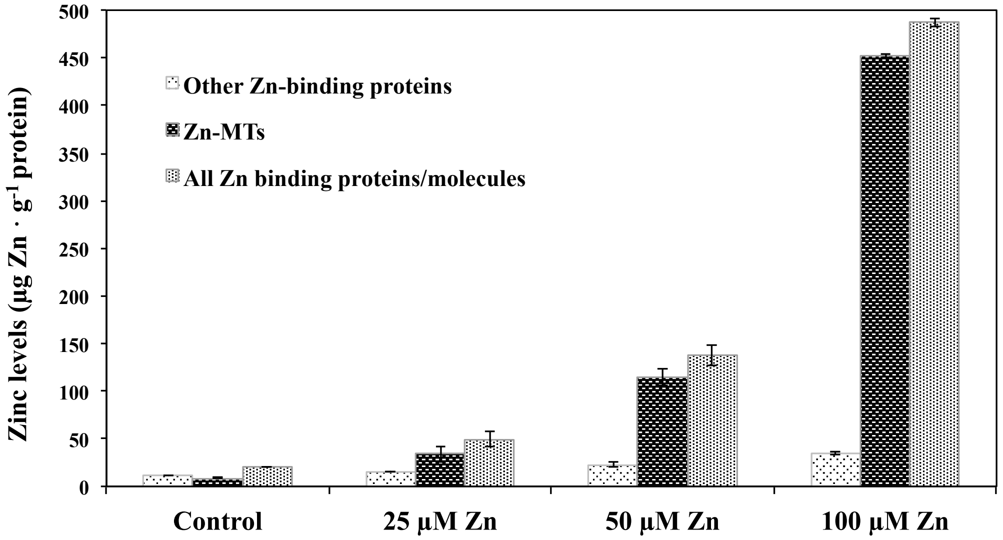

Figure S4. Levels of MTs in non-treated and zinc-treated cells. Concentration of MTs (mg MTs·g^{-1} total protein) labeled with ^{nat}Zn (natural contribution in light grey bars) or ^{68}Zn (exogenous contribution in dark grey bars), obtained by ID-, IPD-, SEC-ICP-MS. HRPEsv cells were either not treated (control) or exposed to $^{68}ZnSO_4$ at 25, 50 or 100 μM separately for 24 h.

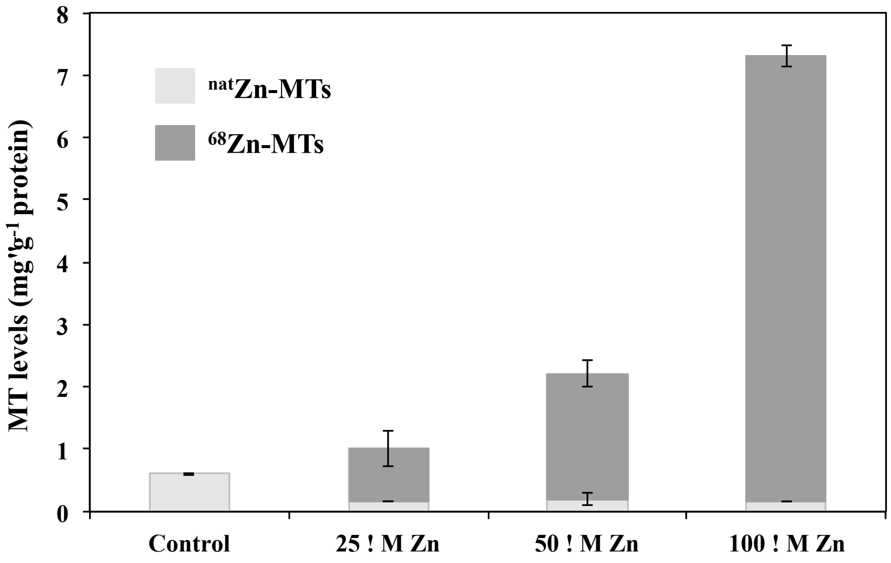

Figure S5. Zinc levels in non-treated or zinc pre-treated and subsequent APPH treated cells. Concentration of zinc (μg Zn·g^{-1} protein) in MTs (black bars dotted), in proteins/biomolecules other than MTs (white bars dotted), and in all Zn-binding proteins/biomolecules (grey bars dotted) obtained by IDA-, IPD-, SEC (HPLC)-ICP-MS in the water-soluble proteins in HRPEsv cells not treated (control) or pretreated with $^{68}ZnSO_4$ at 25, 50, or 100 μM for 24 h and following exposure to 5 mM of APPH for 1 h.

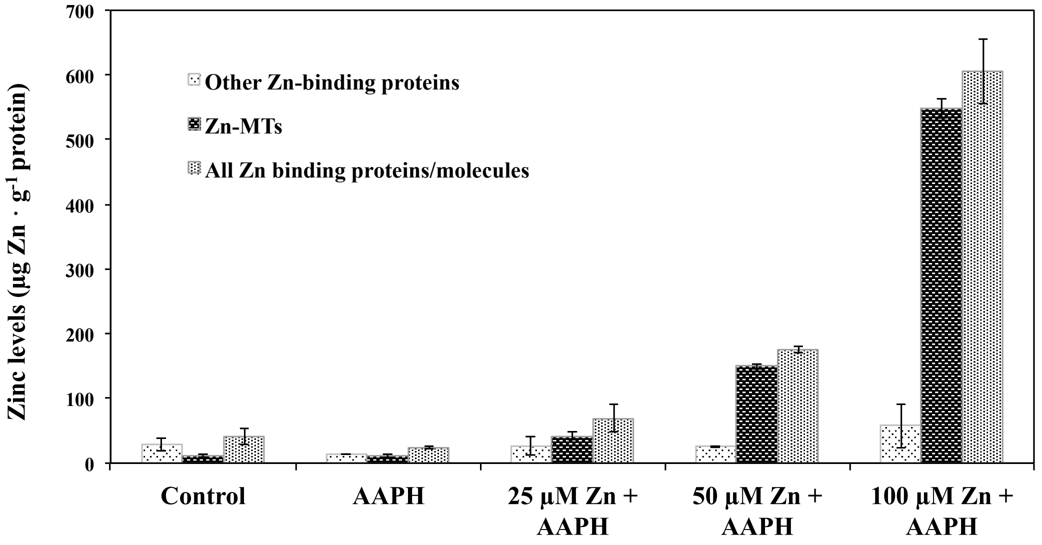

Figure S6. Levels of MTs in non-treated or zinc pre-treated and subsequent APPH treated cells. Concentration of MTs (mg MTs·g^{-1} total protein) labeled with ^{nat}Zn (natural contribution in dark grey bars) or ^{68}Zn (exogenous contribution in dot bars), obtained by ID-, IPD-, SEC-ICP-MS. HRPEsv cells were either not treated (control) or pretreated with $^{68}ZnSO_4$ at 25, 50, or 100 µM for 24 h and followed with an exposure with 5 mM of APPH for 1 h.

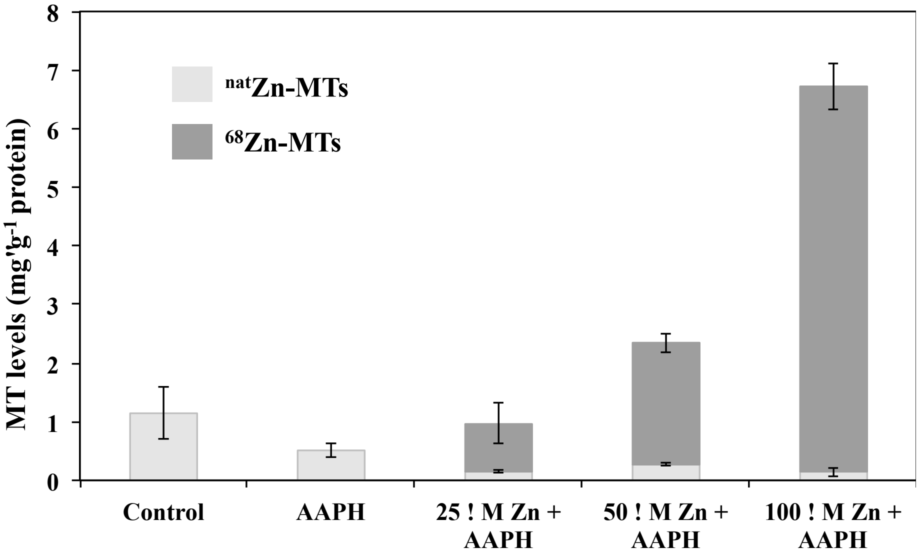

© 2018 by the authors. Submitted for possible open access publication under the terms and conditions of the Creative Commons Attribution (CC BY) license (http://creativecommons.org/licenses/by/4.0/).
